# Representing Quantum Information with Digital Coding Metasurfaces

**DOI:** 10.1002/advs.202001648

**Published:** 2020-09-06

**Authors:** Guo Dong Bai, Tie Jun Cui

**Affiliations:** ^1^ State Key Laboratory of Millimeter Wave Southeast University Nanjing 210096 China; ^2^ Institute of Electromagnetic Space Southeast University Nanjing 210096 China

**Keywords:** classical entanglements, metasurfaces, superposition

## Abstract

With the development of science and technology, the way to represent information becomes more powerful and diversified. Recent research on digital coding metasurfaces has built an alternative bridge between wave‐behaviors and information science. Different from the logic information in traditional circuits, the digital bit in coding metasurfaces is based on wave‐structure interaction, which is capable of exploiting multiple degrees of freedom (DoFs). However, to what extent the digital coding metasurface can expand the information representation has not been discussed. In this work, it is shown that classical metasurfaces have the ability to mimic qubit and quantum information. An approach for simulating a two‐level spin system with meta‐atoms is proposed, from which the superposition for two optical spin states is constructed. It is further proposed that using geometric‐phase elements with nonseparable coding states can induce the classical entanglement between polarization and spatial modes, and give the condition to achieve the maximal entanglement. This study expands the information representing range of coding metasurfaces and provides an ultrathin platform to mimic quantum information.

## Graphical abstract

Here, it is presented that the information represented by digital coding metasurfaces is capable of mimicking the similar functions of qubit and quantum information. The essential viewpoint uses meta‐atoms and metasurfaces to mimic the spin superposition and the classical entanglement between different degrees of freedom. This study provides the feasibility to mimic quantum information with ultrathin platforms.

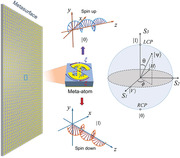

## 1 Introduction

The progress of science and technology has revolutionized the way to represent information. In 1938, Shannon highlighted the analogy between Boolean operations and electronic switching circuits in his pioneering paper.^[^ ^1^ ^]^ Generally, the mathematical operations, defined as binary bits [i.e., 0 (false) and 1 (true)], require only two stable physical states, which can be realized through the use of electronic digital circuits implementing switching elements such as diodes and transistors. This simplicity has played important roles in the birth of digital information age, and made the fields of digital communication and digital signal processing powerful and ubiquitous. Over the past several decades, quantum information science has emerged to achieve the information storage, communication, and computation by harnessing the quantum mechanical effects.^[^ ^2^, ^3^, ^4^, ^5^ ^]^ Through exploiting the superposition principle and entanglement of quantum mechanics, quantum information processing enables large improvements in computational efficiency and communication security.

Since the late 1990s, the artificially constructed structures, termed as metamaterials, have made great progress in manipulating wave behaviors.^[^ ^6^, ^7^, ^8^, ^9^, ^10^, ^11^, ^12^ ^]^ Novel functionalities can be achieved by locally tailoring the electric and magnetic responses of the metastructures, such as negative refraction,^[^ ^6^ ^]^ cloaking,^[^ ^7^, ^8^, ^9^ ^]^ invisible gateway,^[^ ^10^ ^]^ illusion devices,^[^ ^11^, ^12^ ^]^ and metamaterials lens for quantum computation.^[^ ^13^ ^]^ Besides, due to the capability of designing extremely compact devices, the dimension‐reducing metamaterials, dubbed metasurfaces, have attracted great attentions.^[^ ^14^, ^15^, ^16^, ^17^, ^18^, ^19^, ^20^, ^21^, ^22^, ^23^ ^]^ This type of metamaterial provides an avenue to steer electromagnetic (EM) waves through introducing abrupt phase changes on ultrathin interfaces,^[^ ^14^ ^]^ leading to applications involving ultrathin flat lens,^[^ ^15^, ^16^ ^]^ light beam shaping,^[^ ^17^, ^18^ ^]^ skin cloaks, and even quantum entanglement.^[^ ^19^, ^20^, ^21^ ^]^ Furthermore, this promising technology has been applied to some interdisciplinary fields.^[^ ^22^, ^23^ ^]^ The interest towards information processing system has sparked the advent of digital coding metasurfaces, where the subwavelength elements are used to engineer the binary codes.^[^ ^24^ ^]^ New information systems, including direct‐emission communication system,^[^ ^25^ ^]^ multitasking shared aperture,^[^ ^26^ ^]^ reprogrammable holograms,^[^ ^27^ ^]^ and space‐time‐coding metamaterials have been reported.^[^ ^28^, ^29^, ^30^ ^]^

Although the development of digital coding metasurface flourishes the information processing systems, it is still unclear and even not discussed to what extent it will expand the representing ability of information. In this paper, we show that the information represented by digital coding metasurfaces is far beyond the classical information domain, and has the capability of mimicking the function similar to quantum bits or quantum information. We manifest analytically and numerically how a meta‐atom can simulate a two‐level spin system, and represent the superposition of optical spin states. We further present a method to use geometric‐phase elements with nonseparable coding states to formulate the classical entanglement, and derive the requirement to achieve the maximum entanglement. Note that the main idea of the proposed analog is using the classical EM waves to construct similar mathematical structures of the quantum information, not involving the essence of quantum mechanics such as the topic of connotation of probability and nonlocality. This study provides an ultrathin platform to represent quantum information and is helpful to expand the functionalities of metasurfaces.

## 2 Results

### 2.1 Two‐Level Spin System Based on Meta‐Atoms

Qubits are two‐level quantum systems.^[^ ^3^ ^]^ The prototypical two‐level system is an electron spin states, which has been widely used in quantum information processing since the spin Hall effect (SHE) was successfully proved in experiments.^[^ ^31^, ^32^ ^]^ SHE originates from spin–orbit coupling of electrons, resembling the coupling of transverse electric and magnetic components of a propagating EM field.^[^ ^33^ ^]^ Thus, the photonic analogy of spintronics will help the conceptual developments and give rise to novel predictions. A variety of optical analogies of SHE has been reported.^[^ ^34^, ^35^, ^36^, ^37^, ^38^, ^39^ ^]^ In these analogies, the right‐hand circularly polarized (RCP) and left‐hand circularly polarized (LCP) waves correspond to the two spins, and the common phase for classical and quantum physics, i.e., the geometric phase, causes spin split in both momentum and real spaces.

**Figure 1:**
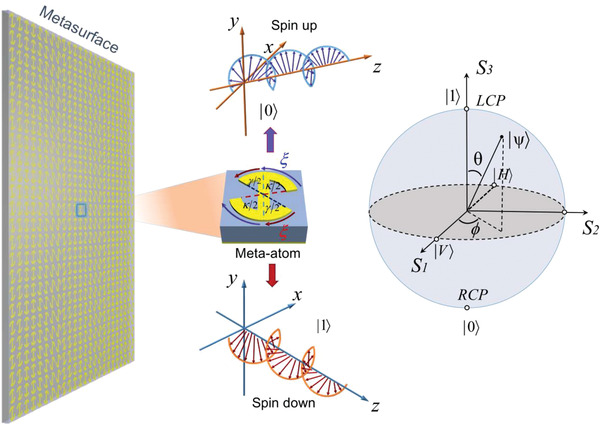
The schematic of representing quantum information with metasurfaces. The meta‐atom is capable of simulating a two‐level spin system. When the spin waves interacting with the meta‐atom, the induced spin currents direct independently along different routes on the structure, causing phase shifts prescribed by the geometric characters of the paths. The interaction between spin waves and the meta‐atom expresses as a weighted superposition of the two optical spin states, which can be intuitively indicated on a Poincare sphere.

In this work, the two‐level spin system is engineered by the meta‐atom shown in **Figure** **1**, which consists of a pair of metal arcs with changing central angles and a connected half‐wave plate. The period of the structure is 7 mm, in which the outer radius of arcs is 3 mm, and the linewidth is 1.0 mm. The structure is printed on a 3 mm thick dielectric board (F4B) (*ε*_r_ = 2.65, *δ* = 0.001) with a metallic ground. When the spin waves are interacting with the structures, the excited spin currents are independent of each other, and direct along specific trajectories on the meta‐atoms, as illustrated in Figure S1 in the Supporting Information. This phenomenon can be regarded as an optical analogy of the Aharonov–Bohm effect, giving rise to corresponding phases prescribed by the geometric characteristics of arcuate paths.^[^ ^40^, ^41^ ^]^ The result of wave‐structure interaction can be expressed by the following equation

(1) $$\begin{array}{c} |\psi 〉=\sigma_{1}e^{-i\chi_{1}}+\sigma_{2}e^{-i\chi_{2}} \end{array}$$

where *σ*_1_ and *σ*_2_ correspond to two spin states, and the phase factors are obtained by the path integral

(2) $$\begin{array}{c} \chi_{1}=\int_{\kappa }^{180^{\circ }}\Omega d\xi ,\;\chi_{2}=\int_{\gamma }^{180^{\circ }}\Omega d\xi \end{array}$$

in which Ω is the varying velocity that relates to the azimuth angle: Ω = d*φ*//d*ξ*. It is clear that the path‐dependent phase appears at a unified geometric phase type, which is only determined by the central angle of the arcuate paths and their initial and final states.^[^ ^34^, ^40^ ^]^ The interference between the two spin states will evidently depend on the phase difference.

### 2.2 State Superposition on the Two‐Level Spin System

Unlike the classical bit that falls into one of the two distinct states, 0 or 1, the qubit can be considered as a weighted superposition of the two orthogonal states

(3) $$\begin{array}{c} |\psi 〉=\alpha |0〉+\beta |1〉 \end{array}$$

in which, the coefficients *α* and *β* are complex numbers. In quantum mechanics, the measured results present with probabilities |*α*|^2^ and |*β*|^2^. Due to the conservation of probability, the sum of the probabilities should be one, satisfying |*α*|^2^ + |*β*|^2^ = 1.

We can rewrite Equation (1) as a state of polarization superposition, performed as

(4) $$\begin{array}{c} |\psi 〉=I_{0}e^{-i\chi_{1}}|0〉+I_{1}e^{-i\chi_{2}}|1〉 \end{array}$$

where |0〉 and |1〉 indicate spin up and down states, *I*_0_ and *I*_1_ are the amplitude coefficients. In this situation, the superposition polarization can be arbitrary state on a Poincare sphere, as shown in Figure 1. The condition for square of the amplitudes will be fulfilled when we measure the far‐field components of the spin states. In this process, the phase term will vanish during the Fourier transform, and the measured results do not present the probability, but present the far‐field energy distribution, which is proportional to the squared amplitudes. Since the meta‐atom generates a perfect reflection efficiency that is higher than 90%, leading to |*I*_0_|^2^ + |*I*_1_|^2^ ≈ 1. Actually, if we only consider the measured results, the sum of the energy distribution can be normalized to 1. As a result, we get the same mathematical expression as the superposition principle.

**Figure 2:**
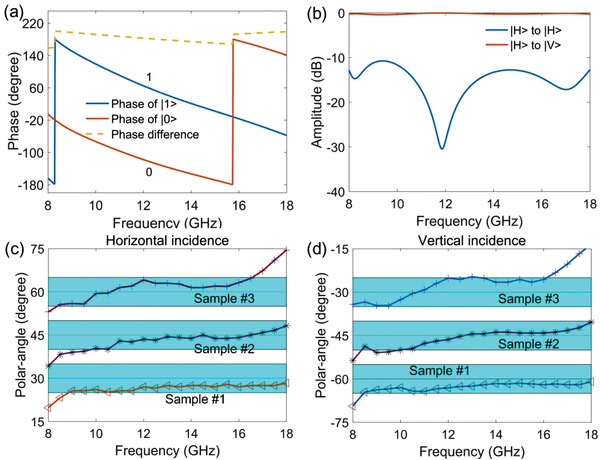
Superposition of optical spins leads to the generation of arbitrary linear polarizations. a) The phase responses of a meta‐atom with the path parameters *κ* = 25° and *γ* = 175°, which imparts the 1‐bit coding states “0” and “1” to the spin states |0〉 and |1〉. b) The above meta‐atom can change the incident polarization |*H*〉 to the output polarization |*V*〉. c) The generated polarizations of three metasurface samples under the incidence of |*H*〉. d) The output polarizations of the three metasurface samples under the incidence of |*V*〉.

In addition to characterize spin superposition, the proposed meta‐atom can make the phase coding more flexibly. In the area of digital coding metasurfaces, 1‐bit coding state is based on two meta‐atoms with 180° phase difference. Generally, this phase difference is obtained under a specific polarization. Here, since the phase factors of spin up and down only depend on their own path parameters, the 180° phase difference can be designed on two spin states. According to Equation (2), this treatment could be accomplished if we set the path parameter of one spin at the start position of its arcuate trajectory, while the parameter of the other spin at the end position of the counterpart route. In actual design of the structure, to ensure the spin currents experiencing arcuate paths with no disturbance, the central angle of the start and end position of the routes are set at 25° and 175°, respectively (see Figure S2 in the Supporting Information for details). In reality, as the spin currents approach to the end of the arcuate trajectories, the coupling will be enhanced, so as to increase the phase difference. Consequently, when *κ* = 25° and *γ* = 175°, the phase difference of the two spins can achieve 180°, as shown in **Figure** **2**a. Therefore, we impart the 1‐bit coding states “0” and “1” to the spin states |0〉 and|1〉. For convenience, in the following, the phase coding states are represented with numbers, and the polarization states (and the spatial modes in the next section) are indicated in bracket. Under above circumstance, the incident horizontal (|*H*〉) polarization will be changed to vertical (|*V*〉) polarization by the meta‐atom, as shown in Figure 2b. If we set *κ = γ*, the structure manifests a symmetric geometric configuration, and the phase difference is always 0°, meaning the coding states are both “0” for the two spins, the output polarization remains as |*H*〉, illustrated in Figure S3 in the Supporting Information.

We have discussed the superposition states of two special cases in above contents. It is better to consider the more general situation when simulating the similarities of quantum bit. If we fix the spin‐up trajectory at the start position, while the trajectory of spin down evolving from start to end, the phase difference of two spins will vary from 0° to 180°, causing phase coding states falling into the interval between “0” and “1.” In this regard, the superposition state can be arbitrary linear polarization, which is a state expanded by |*H*〉 and |*V*〉, and can be simply deduced from Equation (1)

(5) $$\begin{array}{c} |\psi 〉=\cos\frac{\Delta \chi }{2}|H〉+\sin\frac{\Delta \chi }{2}|V〉 \end{array}$$

where ∆*X* is the phase difference, depending on the path parameters

(6) $$\begin{array}{c} \Delta \chi =\chi_{2}-\chi_{1}=\gamma -\kappa \end{array}$$

Ultimately, the output linear polarization angle is (*γ* − *κ*)/2. More importantly, in Equation (5), the sum of the squared amplitudes naturally equals 1, meeting the requirement of superposition principle.

To demonstrate the capability of the proposed method, three different linear polarizations are generated with metasurfaces. As described, the meta‐atoms are designed with fixed *κ* and varying *γ*, the detailed parameters can be seen in **Table** **1**, and geometric configurations of the structures are shown in Figure S4 in the Supporting Information. Then, each of the meta‐atoms is arranged along the *x* and *y* directions, engineering three 31 × 31 arrays. We use the commercial software, CST Microwave Studio, to calculate the far‐field results of the metasurfaces. In simulations, the metasurfaces are excited by horizontally polarized plane waves. According to Equation (6), the generated polarization angles of the three metasurfaces are 30°, 45°, and 60°, respectively, as shown in the fourth column in Table 1. The simulated results are sketched in Figure 2c, where the polarization angles are obtained by $\tan^{-1}\left(\sqrt{w_{y}/w_{x}}\right)$, *w_x_* and *w_y_* denote the *x* and *y* components of the far field. The designed metasurfaces work well, from which the generated polarizations direct to the target angles within ±5° error range under a broad bandwidth from 9 to 16 GHz. The far‐field results support the viewpoint that the meta‐atom can realize the superposition state of two optical spins. Actually, the generated polarization can be modified as ${\left(\cos\left(\frac{\Delta \chi }{2}\right),\sin\left(\frac{\Delta \chi }{2}\right)\right)}^{T}e^{i(\frac{\kappa +\gamma }{2})}$, coinciding the formula of the spin states moving in a circular routes, where the reference phase factor $e^{i(\kappa +\gamma )/2}$ can be regarded as the consequence of certain gauge transformation. Besides, from the perspective of application, the designed metasurfaces have the ability to rotate arbitrarily linear polarizations. Under the incidence of |*V*〉, the three samples will rotate the polarization angle to −60°(120°), −45°(135°), and −30°(150°), respectively, as seen in the last column in Table 1. Figure 2d gives numerical results, matching very well with the theoretical analyses.

**Table 1:** Three metasurface samples and the generated polarizations

Meta‐atom			Polar‐angle [°]	
Path parameter	*γ* [°]	*κ* [°]	Horizontal	Vertical
Sample # 1	85	25	30	−60
Sample # 2	115	25	45	−45
Sample # 3	145	25	60	−30

It is well known that qubit has uncertainty, which does not exist in the classical physics. In the proposed analog, if the central angle of the arcuate paths on the meta‐atom is fixed, the output polarization will be determined. However, if the spin trajectories can be dynamically controlled, the output polarization will vary with the evolution of geometric phase difference. The microfluidic technology is a good candidate to achieve such an active control process, where the arcuate paths can be implemented by liquid metal injecting through a pneumatic control system.^[^ ^42^ ^]^ Under this circumstance, the output linear polarization can be continuously adjusted from |*H*〉 to |*V*〉. Further, we may manipulate the arcuate trajectories in an irregular way, giving rise to the output polarization states behaving as specific probability density distribution. As a result, we can use the active two‐level spin system to realize the similar property as the uncertainty. We remark that significant difference exists between the proposed meta‐atom and the anisotropic metastructures for polarization conversion. For the anisotropic metastructures, the output polarization usually depends on different EM responses in two orthogonally linear polarizations. Here, the proposed meta‐atom is a geometric‐phase element. When the linearly polarized wave interacts with the meta‐atom, the output state is coherent superposition of two spin states, which depends on their phase difference and can be modulated by manipulating the arcuate trajectories. Since the output polarization can be expanded by |*H*〉 and |*V*〉, it is feasible to use the method of anisotropic metasurfaces to analyze the above three samples and obtain the correct results. However, the intrinsic mechanism of the polarization conversion relies on the evolution of geometric phases.

### 2.3 Nonseparable Coding States and the Induced Classical Entanglement

Entanglement is one of the most fascinating characteristics in quantum mechanics and has significant application in quantum information.^[^ ^3^ ^]^ The essential property of entanglement is nonseparability, which means that a state of a composite system cannot be expressed as tensor product of its subsystems.^[^ ^3^, ^43^, ^44^ ^]^ There are two types of entanglement: intersystem entanglement and intrasystem entanglement.^[^ ^43^, ^44^ ^]^ The intersystem entanglement only occurs in separated particles, such as the entanglement of EPR pair.^[^ ^45^, ^46^ ^]^ In this case, the measurement of the state of one particle in the two‐particle entangled state can define the state of the other particle instantaneously, regardless of their spatial separation. This phenomenon is a manifestation of quantum nonlocality. Thus, the intersystem entanglement is also called as nonlocal entanglement. Conversely, the intrasystem entanglement investigates the relation between different DoFs of a single system, showing the local nonseparability.^[^ ^43^, ^44^ ^]^ For example, the local entanglement between spatial and spin DoFs in single neutron belongs to this category.^[^ ^47^ ^]^ The intrasystem entanglement can find counterparts in the classical physics, called as classical entanglement. By exploiting the nonseparability of classical systems, the classical entanglement is capable of constructing the mathematical structures equivalent to the formula exhibited by the quantum entangled states. This concept was firstly introduced into the classical optics by Spreeuw^[^ ^43^ ^]^ where he pointed out that the nonseparable relation between polarization and spatial DoFs of light can present the correlation like entanglement. Since then, a variety of studies have been devoted to search for the nonseparable relationship between different classical DoFs.^[^ ^44^, ^48^, ^49^, ^50^, ^51^, ^52^, ^53^, ^54^, ^55^, ^56^ ^]^ Most approaches to establish the classical entanglement in optics are based on the polarization and other DoFs, such as the polarization and spatial modes,^[^ ^43^, ^44^, ^48^, ^49^, ^50^, ^51^, ^52^ ^]^ the polarization and frequencies,^[^ ^53^ ^]^ as well as the polarization and orbital angular momentum (OAM) modes.^[^ ^54^ ^]^ Although the nonlocality does not exist in these analogs of classical entanglement, they are proving to be violation of the Bell inequality,^[^ ^51^, ^52^ ^]^ and are useful tools to simulate quantum algorithms and metrology.^[^ ^55^, ^56^ ^]^

The key to establish classical entanglement is finding the inseparable correlation between different DoFs. We point that nonseparability exists in the phase values of geometric‐phase elements, from which the nonseparable coding states for two spins are produced. When the proposed meta‐atom is rotating, the Pancharatnam–Berry (PB) phase will be generated. Suppose the rotating angle is *τ*, the PB phase profile behaves as*φ*_R_ = −*φ*_L_ = 2*τ*, leading to the spin states present a conjugate relationship. Therefore, the interaction between spin waves and metastructure can be described as

(7) $$\begin{array}{c} |\psi 〉=\sigma_{1}e^{-i\left(\chi_{1}+2\tau \right)}+\sigma_{2}e^{-i\left(\chi_{2}-2\tau \right)} \end{array}$$

**Figure 3:**
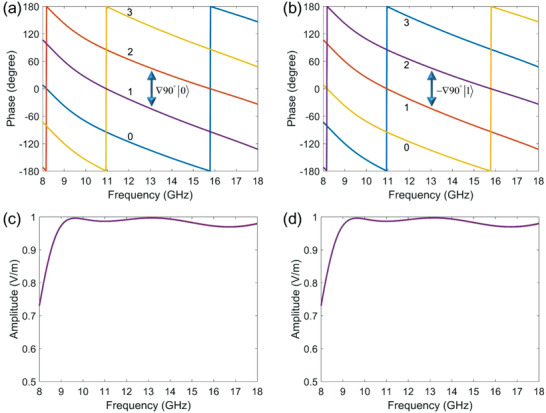
The nonseparable coding states with one‐to‐one mapping correlation. a,b) Phase responses of the coding meta‐atoms on two spin states. Note that the phase responses of a specific meta‐atom on two spins are indicated with the same color curves. The coding states for two spins perform nonseparable, when the meta‐atoms impart codes “0,” “1,” “2,” “3” on |0〉, the coding states “3,” “2,” “1,” “0” will be naturally assigned on |1〉. c,d) Amplitude responses of the coding meta‐atoms, which shows high efficiency under a broad bandwidth. As the coding meta‐atoms are obtained by a single structure via geometric transformations, no parameter changes, the amplitude responses are exactly same.

Operating simple geometric transformations onto the proposed meta‐atoms can generate desired phase profiles. The geometric configurations of the selected meta‐atoms are presented in Figure S5 in the Supporting Information. Firstly, we take Sample #2 in Table 1 as the referenced coding state. Carrying out an axial symmetry transformation on the structure, the path parameter will change to *γ* = 25° and *κ* = 115°. According to Equation (2), the phase factor of spin up decreases 90°, and the counterpart phase of spin down increases 90°. Then rotate the transformed structure with *τ* = 90°, forming the third meta‐atom. Based on Equation (7), this operation will impose ±90° phase shifts relatively to the referenced phase values. The fourth structure is obtained by rotating Sample #2 with 90°. In this instance, the PB phase will bring ±180° phase shifts for two spins. Actually, the second meta‐atom can be regarded as generating ±270° relative phase shifts. We create the coding states by virtue of the phase values. The four structures build 2‐bit coding states for two spins, in which the coding states are always nonseparable. That is to say, when the structures impart codes “0,” “1,” “2,” “3” on |0〉, the coding states “3,” “2,” “1,” “0” will be naturally assigned on |1〉. The simulated phase responses for the above meta‐atoms are shown in **Figure** **3**a,b, where the numerical results agree with the theoretical analyses very well, and we notice that the ∇ ± 90° gradient phase profiles for two spins are engineered. Figures 3c,d present the corresponding amplitude responses. Because Sample #2 only experiences the geometric transformations, no parameter changes, thus the amplitude responses of the four meta‐atoms are exactly same. Apparently, the amplitudes are all higher than 0.95 in a broad bandwidth. Hereafter, we will show how to generate classical entanglement between polarization and spatial modes by utilizing the nonseparable coding states. It should be noted that, because the observed objects are far‐field beams or field profiles, the phase information cannot be measured directly but can be deduced by the scattering theory.

**Figure 4:**
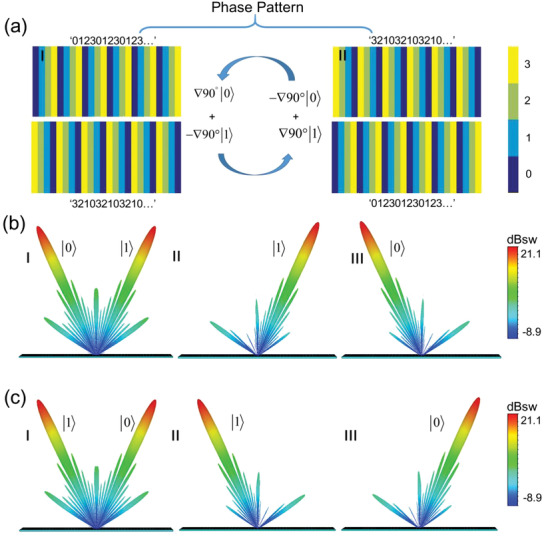
The induced maximal entanglement between polarizations and spatial modes. a) The two categories of coding patterns, in which the coding pattern in (II) can be obtained by flipping the pattern in (I). b) The far‐field scattering pattern of the coding pattern in (aI). c) The far‐field scattering pattern of the coding pattern in (aII), the result is symmetric with the scattering pattern in (b).

Introducing the design concept of coding metasurfaces can manipulate the polarization and spatial modes easily. Due to the nonseparability, the encoding patterns for the two spins always relates to each other, as shown in **Figure** **4**a. In Figure 4a I, the coding sequence for spin up is “012301230123…,” meanwhile, the spin‐down coding sequence is automatically “321032103210….” The coding pattern in Figure 4a II is obtained by flipping the pattern in Figure 4a I, where the gradients of the coding sequences for the spin states are still opposite to each other. We implement the above coding patterns by using the 2‐bit coding structures, in which every coding unit is a supercell compromising of two identical meta‐atoms. The scattering patterns of the metasurfaces under the horizontal polarization is a nonseparable form of spin states and spatial modes, which is

(8) $$\begin{array}{c} \Psi =P_{1}S_{1}\left(\theta ,\varphi \right)+P_{2}S_{2}\left(\theta ,\varphi \right) \end{array}$$

where (*P*_1_, *P*_2_) and (*S*_1_, *S*_2_) are the orthonormal bases for polarization and far‐field spatial modes, respectively. The spin states are naturally orthogonal, and the orthogonal condition for the spatial modes is satisfied when the far‐field beams are not overlapping. Equation (8) displays a classical entanglement between polarizations and spatial modes, which has the same mathematical structure of a 2‐qubit entangled state,^[^ ^44^ ^]^ and can be written as a Schmidt decomposition with the following form^[^ ^44^, ^57^ ^]^

(9) $$\begin{array}{c} |\Psi 〉=\sqrt{\mu_{1}}|P_{1}〉|S_{1}〉+\sqrt{\mu_{2}}|P_{2}〉|S_{2}〉 \end{array}$$

If *μ*_1_ *=* 0 or *μ*_2_ *=* 0, the state is factorable and the spins and spatial modes are independent. Conversely, if *μ*_1_, *μ*_2_ *≠* 0, then |Ψ〉 is entangled. In this manner, the amount of entanglement can be quantified by the Schmidt number *K* defined as

(10) $$\begin{array}{c} K=\frac{{\left(\mu_{1}+\mu_{2}\right)}^{2}}{\mu_{1}^{2}+\mu_{2}^{2}} \end{array}$$

*K =* 1 denotes the factorable state, and *K =* 2 means the maximal entanglement appearing when *μ*_1_ *= μ*_2_.

Theoretically, the scattering patterns for the metasurfaces of Figure 4a are

(11) $$\begin{array}{c} \left\{\begin{array}{c} |\Psi_{1}〉=\frac{1}{\sqrt{2}}|0〉|\left(-\sin^{-1}\;\left(\frac{\lambda }{\Gamma }\right),180^{\circ }\right)〉+\frac{1}{\sqrt{2}}|1〉|\left(\sin^{-1}\;\left(\frac{\lambda }{\Gamma }\right),0^{\circ }\right)〉\\ |\Psi_{2}〉=\frac{1}{\sqrt{2}}|1〉|\left(-\sin^{-1}\;\left(\frac{\lambda }{\Gamma }\right),180^{\circ }\right)〉+\frac{1}{\sqrt{2}}|0〉|\left(\sin^{-1}\;\left(\frac{\lambda }{\Gamma }\right),0^{\circ }\right)〉 \end{array}\right. \end{array}$$

where *λ* is the working wavelength, and *Γ* is the period of the metasurfaces. The Schmidt number *K* for the two cases are 2, indicating that the two spins and spatial modes present the maximal entanglement. In other words, when the spin‐up state is measured at the position (sin ^−1^(*λ*/Γ),0), the measured state at the symmetric position must be the spin down, and vice versa.

The numerical simulation results in Figure 4b,c have verified the theoretical analyses. The working frequency of the metasurfaces is selected at 13 GHz, and the period *Γ* is 56 mm. Figure 4b shows the far‐field scattering pattern of Figure 4a I, in which the spin‐up and spin‐down beams direct to the spatial modes of (−24.3°, 180°) and (24.3°, 0°), agreeing well with the theory prediction (−24.4°, 180°) and (24.4°, 0°). Figure 4c gives the far‐field results of the coding pattern of Figure 4a II, where all the components are symmetric with the cases of Figure 4b. The numerical results of Schmidt number *K* for the two cases are calculated as 2, validating the maximal entanglement.

### 2.4 Nonmaximal Entanglement Induced by Nonseparable Coding States with One‐To‐Multiple Mapping

**Figure 5:**
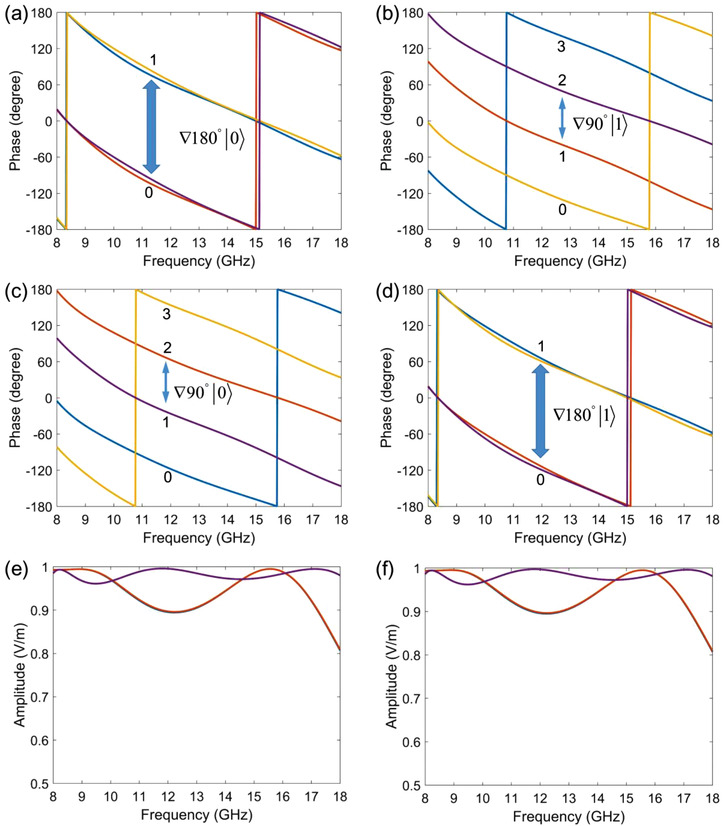
The nonseparable coding states with one‐to‐multiple mapping correlation. a,b) Phase responses of the coding states, which can generate 1‐bit coding states for spin up and 2‐bit coding states for spin down. c,d) Phase responses of the other group of coding states, which have the symmetric geometric configuration with the above coding meta‐atoms, and generate 2‐bit coding states for spin up and 1‐bit coding states for spin down. e,f) Amplitude responses of the coding states are higher than 0.9 in a broad bandwidth. Since the coding states are obtained by two structures via geometric transformations, the amplitude responses overlap into two curves.

The reason of the polarization and spatial modes achieving the maximal entanglement stems from the nonseparable coding states carrying single mapping relationship, i.e., each coding state of spin up only has a single counterpart coding state of spin down, and vice versa. It is considered that the entanglement effect will be affected if the one‐to‐one mapping is broken. Meticulously adjusting the trajectories of spin currents on the meta‐atoms may achieve the needed phase profiles. To break the single mapping relationship, we could impart 2‐bit coding states to one spin, and assign 1‐bit coding states on the other spin. The purpose can be realized by several simple geometric transformations. First, we should let either of the spin paths maintain fixed. For example, we set the spin‐up trajectory remain at the final position *κ* = 175°, and make the spin‐down path parameter evolve with *γ =* 25*°* and *γ =* 115*°*. Under this strategy, the spin‐up phase shifts will be fixed at one factor, nevertheless, the spin‐down phase shifts will generate a −90° phase difference. Then we rotate the two meta‐atoms with *τ =* 90*°*, the phase shifts of spin up will increase 180°, changing to another fixed factor. In the meanwhile, the spin‐down phase shifts generate −180° and −270° phase differences relative to the referenced phase value. Consequently, we have built 1‐bit coding states for spin up, and 2‐bit coding states for spin down, as shown in **Figure** **5**a,b. The coding states will exchange when we maintain the spin‐down trajectory fixed and carry out the above steps, as illustrated in Figure 5c,d. The geometric configurations of the two groups of meta‐atoms can be seen in Figure S6 in the Supporting Information. Evidently, the coding states for two spins are still nonseparable, but the mapping changed to one‐to‐multiple relationship. The amplitude responses perform high efficiency, as observed in Figure 5e,f.

**Figure 6:**
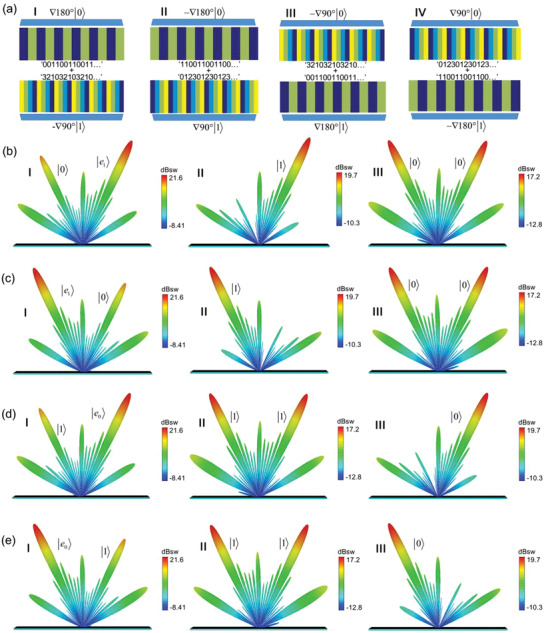
The induced nonmaximal entanglement by nonseparable coding states with one‐to‐multiple mapping. a) Four coding patterns. I) Consists of a periodical sequence “001100110011…” for spin up and a gradient sequence “321032103210…” for spin down. II) Flipping the pattern of (I). III) A spin symmetry distribution of (I). IV) Flipping the pattern of (III). b) The simulated far‐field scattering pattern of the coding pattern in (aI), in which the scattering pattern in (bI) is the superposition of the cases in (bII) and (bIII). c) The far‐field scattering pattern of the coding pattern in (aII), which is symmetric with the result in (b). d) The far‐field scattering pattern of the coding pattern in (aIII), which is a spin symmetric distribution of the cases of (b). e) The far‐field scattering pattern of the coding pattern in (aIV), which is symmetric with the result in (d).

There are four kinds of coding patterns for the one‐to‐multiple coding states, as shown in **Figure** **6**a. The coding pattern in Figure 6a I comprises with a periodical sequence “001100110011…” for spin up and a gradient sequence “321032103210…” for spin down. Flipping the above pattern can get the coding pattern in Figure 6a II. The coding pattern in Figure 6a III consists of a gradient sequence “321032103210…” for spin up and a periodical sequence “001100110011…” for spin down, which is a spin symmetry distribution for the pattern in Figure 6a I. The coding pattern in Figure 6a IV is acquired by flipping the pattern in Figure 6a III. Then the coding patterns are filled with the proposed nonseparable coding states, forming four metasurface samples.

The far‐field scattering patterns of the above four cases under horizontal polarization are more complicated. Assuming the energy of the side lobes are much lower than that of the main beams, the scattering patterns can be expressed as

(12) $$\begin{array}{c} \left\{\begin{array}{c} |\Psi_{1}〉=\frac{1}{2}|0〉|\left(-\sin^{-1}\left(\frac{\lambda }{\Gamma }\right),180^{\circ }\right)〉+\left(\frac{1}{2}|0〉+\frac{1}{\sqrt{2}}|1〉\right)|\left(\sin^{-1}\left(\frac{\lambda }{\Gamma }\right),0^{\circ }\right)〉\\ |\Psi_{2}〉=\frac{1}{2}|0〉|\left(\sin^{-1}\left(\frac{\lambda }{\Gamma }\right),0^{\circ }\right)〉+\left(\frac{1}{2}|0〉+\frac{1}{\sqrt{2}}|1〉\right)|\left(-\sin^{-1}\left(\frac{\lambda }{\Gamma }\right),180^{\circ }\right)〉\\ |\Psi_{3}〉=\frac{1}{2}|1〉|\left(-\sin^{-1}\left(\frac{\lambda }{\Gamma }\right),180^{\circ }\right)〉+\left(\frac{1}{2}|1〉+\frac{1}{\sqrt{2}}|0〉\right)|\left(\sin^{-1}\left(\frac{\lambda }{\Gamma }\right),0^{\circ }\right)〉\\ |\Psi_{4}〉=\frac{1}{2}|1〉|\left(\sin^{-1}\left(\frac{\lambda }{\Gamma }\right),0^{\circ }\right)〉+\left(\frac{1}{2}|1〉+\frac{1}{\sqrt{2}}|0〉\right)|\left(-\sin^{-1}\left(\frac{\lambda }{\Gamma }\right),180^{\circ }\right)〉 \end{array}\right. \end{array}$$

in which the second terms on the right hand sides represent the superposition states, which are elliptical polarizations combined by two spins with different amplitudes. The superposition states in *Ψ*_1_ and *Ψ*_2_ are left‐hand elliptical polarizations, while the states in *Ψ*_3_ and *Ψ*_4_ are right‐hand elliptical polarizations. The ellipticity angles of the superposition polarizations are ±*a*tan^−1^(0.17), where ± refers to left and right handedness. For simplicity, we indicate the right‐hand and left‐hand elliptical polarizations as |*e*_0_〉 and |*e*_1_〉. Equation (12) describes complex classical entanglements, the spatial modes remain unaltered, but the polarization bases have been changed to (|0〉, |*e*_1_〉) and (|1〉, |*e*_0_〉), where the handedness of spins and elliptical polarizations are opposite. Under the condition, we cannot recognize the complete scattering pattern by one step measurement. For example, if the spin‐up state are measured at the position (sin ^−1^(*λ*/Γ),0°), we cannot ensure whether the spin‐down state occur at the same position or the symmetric position( − sin ^−1^(*λ*/Γ),180°), further measurement with the spin‐down state or the measured results in the symmetric position are needed. This is because the mapping of coding states between two spins is not one‐to‐one but one‐to‐multiple relationship. The Schmidt number *K* in these cases is equal to 1.6 theoretically, having obvious decrease.

The numerical simulation results are shown in Figure 6b–e, which correspond the far‐field scattering patterns for the coding patterns of Figure 6a I–IV orderly. The selected working frequency and period of the metasurfaces are 12.5 GHz and 56 mm. The scattering pattern in Figure 6b I is the superposition of the cases in Figure 6b II,III, and the elliptical polarization |*e*_1_〉 is synthesized by the beams in the right side of the normal plane, agreeing with the description of *Ψ*_1_. The deflecting angles of the simulated results for |0〉 and |*e*_1_〉 are −25.3° and 25.2°, which are very close to the theoretical results ±23.2°. And the beam energy of |0〉 is −2.5 dB lower than that of |1〉, approaching to the theoretical value −3 dB. The simulated results in Figure 6c–e are also in line with the prediction of Equation 12. For a better quantitative illustration, the values of numerical and theoretical results, represented before and after “/” respectively, are summarized in **Table** **2**. In order to calculate the coefficients of Schmidt decomposition, we sum the energy of the largest five beams in the far‐field region and normalize it to 1. Thus, the results of *μ_1_* + *μ_2_* in simulation is lower than 1. We can observe that the simulated deflected angles, energy distribution, and Schmidt numbers, match well with the theoretical results. Evidently, the numerical results support the viewpoint that the nonseparable coding states with one‐to‐multiple mapping will affect the induced entanglement.

**Table 2:** Comparison of the simulated and theoretical results for the nonmaximal entanglement

Sim.	Deflection angles		Weight coefficients		*K*
The.	Spin states	Superposition	*μ*_1_	*μ*_2_	
*Ψ*_1_	−25.3°/−23.2°	25.2°/23.2°	0.23/0.25	0.63/0.75	1.64/1.60
*Ψ*_2_	25.2°/23.2°	−25.3°/−23.2°	0.63/0.75	0.23/0.25	1.64/1.60
*Ψ*_3_	−25.2°/−23.2°	25.2°/23.2°	0.23/0.25	0.63/0.75	1.64/1.60
*Ψ*_4_	25.2°/23.2°	−25.2°/−23.2°	0.63/0.75	0.23/0.25	1.64/1.60

## 3 Discussion and Conclusion

We have discussed the information representing ability of digital coding metasurfaces, and pointed out that coding metasurfaces are capable of mimicking the similar features of quantum information. The proposed meta‐atoms can simulate two‐level spin systems and generate the superposition states of two optical spins. We have utilized the geometric‐phase elements with nonseparable coding states to induce the classical entanglement, and demonstrated that the maximal entanglement appears when the mapping of the coding states between two spins is a one‐to‐one correlation. Note that the spin states and geometric phases are shared in both quantum and classical fields, thus the analogy has certain physical meanings. We remark that the proposed analog is not the only way to achieve the representation of quantum information. More orthogonal components of EM waves, such as frequencies and OAM modes, can be dynamically connected with the polarizations, simulating the characteristic of qubit and creating multidimensional classical entangled states. In order to construct the similar mathematical structure to the quantum information, the observed physical quantity is far‐field energy distributions or beam properties. If there are a lot of side lobes in the far‐field region, the simulating result will be affected. Recently, some metasurfaces have been introduced into the quantum optics, manifesting powerful capabilities to manipulate multiphoton and high‐dimensional entanglement.^[^ ^21^, ^58^ ^]^ In this case, most of the noise is attributed to the side lobes, which imposes a fundamental limitation to the system's information capacity. Therefore, it is important to design high‐efficiency metasurfaces for the quantum optics. In fact, dielectric metasurfaces can completely avoid the absorption losses. Through optimizing the geometry and arrangement of meta‐elements, the dielectric metasurfaces can suppress the side lobes and realize high efficiency close to 100%,^[^ ^21^, ^59^, ^60^ ^]^ providing promising platform for the quantum information. Last but not least, the constructed classical entanglement here has no nonlocality, and the common property of the classical and quantum entanglements is the nonseparability. The nonlocality of quantum remains an open question.

As a summary, we have extended the information representation of the digital coding metasurfaces. We have demonstrated analytically and numerically how the state superposition principle and the classical entanglement can be implemented using the metasurfaces. This study provides a useful platform for representing the quantum information, and may have potential applications for simulating quantum algorithms and quantum information processing.

## Conflict of Interest

The authors declare no conflict of interest.

## Supporting information

Supporting Information
