# Supplementary material for: Representing Quantum Information with Digital Coding Metasurfaces
Source: Adv Sci (Weinh). 2020 Sep 6;7(20):2001648. doi: 10.1002/advs.202001648 (PMC7578880; doi:10.1002/advs.202001648)
Supplement: Supplementary file 1 — Supporting Information [file ADVS-7-2001648-s001.pdf]

© 2020 Wiley-VCH GmbH

ADVANCED SCIENCE
Open Access

Supporting Information

for *Adv. Sci.*, DOI: 10.1002/advs.202001648

Representing Quantum Information with Digital Coding Metasurfaces

*Guo Dong Bai and Tie Jun Cui**

Supporting Information

Representing Quantum Information with Digital Coding Metasurfaces

*Guo Dong Bai and Tie Jun Cui**

Dr. G. D. Bai and Prof. T. J. Cui
State Key Laboratory of Millimeter Wave, Southeast University, Nanjing, 210096, China

Dr. G. D. Bai and Prof. T. J. Cui
Institute of Electromagnetic Space, Southeast University, Nanjing, 210096, China
E-mail: tjcui@seu.edu.cn

Keywords: metasurface, superposition, classical entanglement

The file includes:

- **Figure S1.** The interaction between spin waves and the meta-atom.
- **Figure S2.** Illustrating the selection of special path parameters with four meta-atoms.
- **Figure S3.** The amplitude response of meta-atom in Figure S2(d).
- **Figure S4.** The three structures that correspond to the parameters in Table 1.
- **Figure S5.** The geometric configuration of meta-atoms, from which the non-separable coding states with one-to-one mapping correlation can be generated.
- **Figure S6.** The two group of meta-atoms, which generate the non-separable coding states with one-to-multiple mapping correlation.

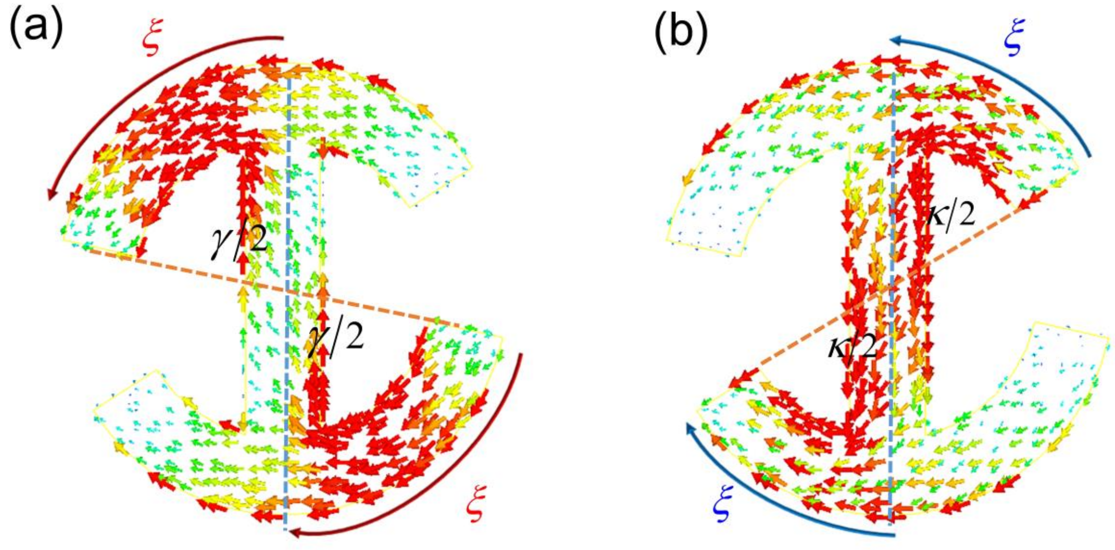

Figure S1. The interaction between spin waves and the meta-atom. (a) The route of spin-down current. (b) The route of spin-up current.

When the spin waves interact with the meta-atom, the induced spin currents direct independently along different routes on the structure, causing phase shifts prescribed by the geometric character of the paths. This path-dependent phase can be explained by the optical Coriolis effect. As the currents on the half-wave plate does not invoke the Coriolis effect, the phase shift of spin states only depends on the arcuate routes. Therefore, we can manipulate the phase profiles of two spins by regulating the path parameters of the corresponding trajectories. As illustrated in Figure S1, the phase shifts of spin up can be modulated by controlling the path parameter κ, while the spin-down phase shifts are determined by γ.

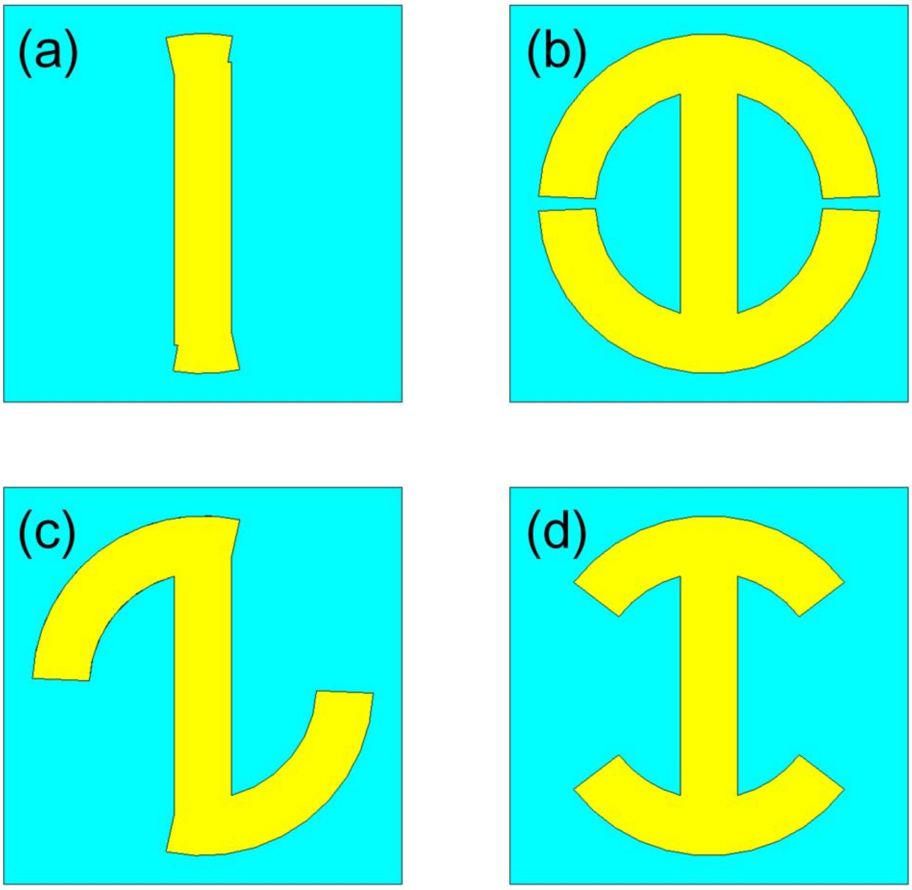

Figure S2. Illustrating the selection of special path parameters with four meta-atoms. The parameters of the four structures are (a) κ = 20° and γ = 25°, (b) κ = γ =175°, (c) κ = 25° and γ =175°, (d) κ = 75° and γ = 75°.

Figure S2 gives four structures as instances to illustrate the selection of path parameters of the trajectories. Figure S2a shows the initial of the trajectories, in which we set κ = 20° and γ = 25°. Clearly, the structue is not complete when κ = 20°, and the defect may cause parasitic effect and affect the routes of current flow. Thus, the central angle of start of the trajectories sets at 25°. Figure S2b sketches the final of the trajectories, if the chosen parameters are bigger than this situation, the two metal arcs will generate strong coupling or connect with each other. As a consequence, the parameter of end of the routes sets at 175°. In Figure S2c, the trajectories for spin up and down are set at the start and end positions, respectively. The structure will generate 180° phase difference of two spin states and change the incident horizontal polarization to vertical polarization, as shown in **Figure 2**a,b in the maintext. Figure S2d shows a symmetric structure, in this case, the phase factors of two spins are always same, and the output remains as horizontal polarization, as shown in the following **Figure S3**.

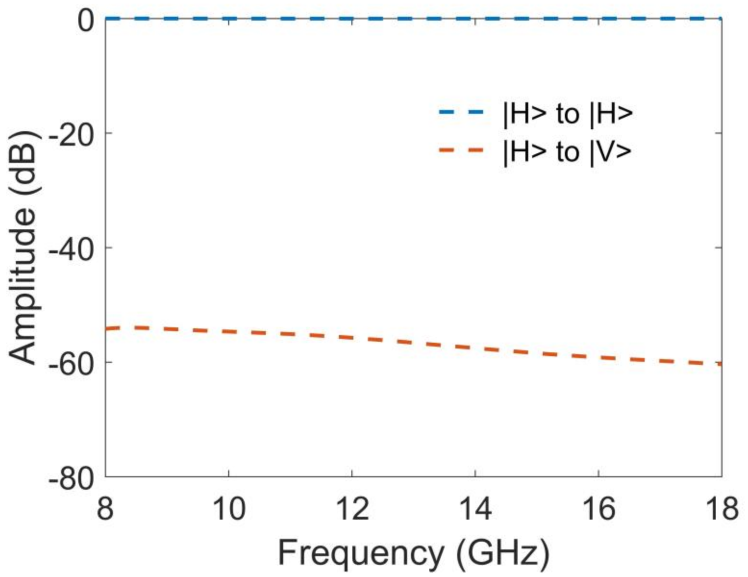

Figure S3. The amplitude response of meta-atom in Figure S2(d). Evidently, the symmetrical structure does not change the polarization states.

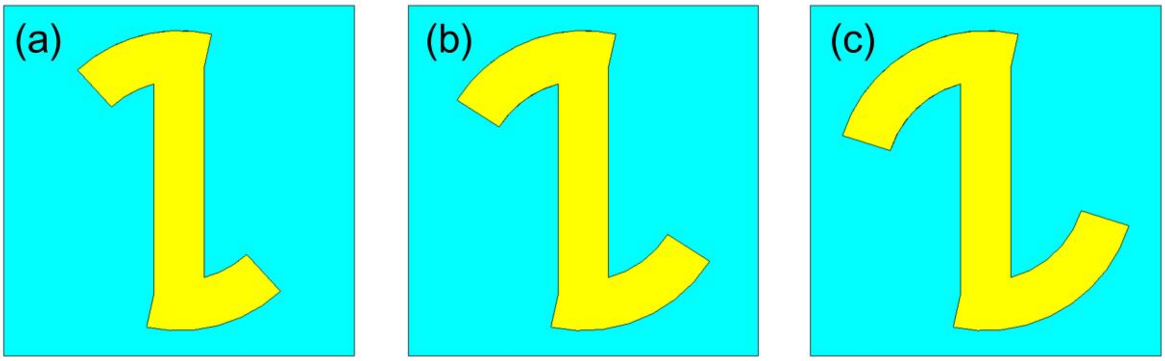

Figure S4. The three structures that correspond to the parameters in Table 1. (a) Sample # 1. (b) Sample # 2. (c) Sample # 3.

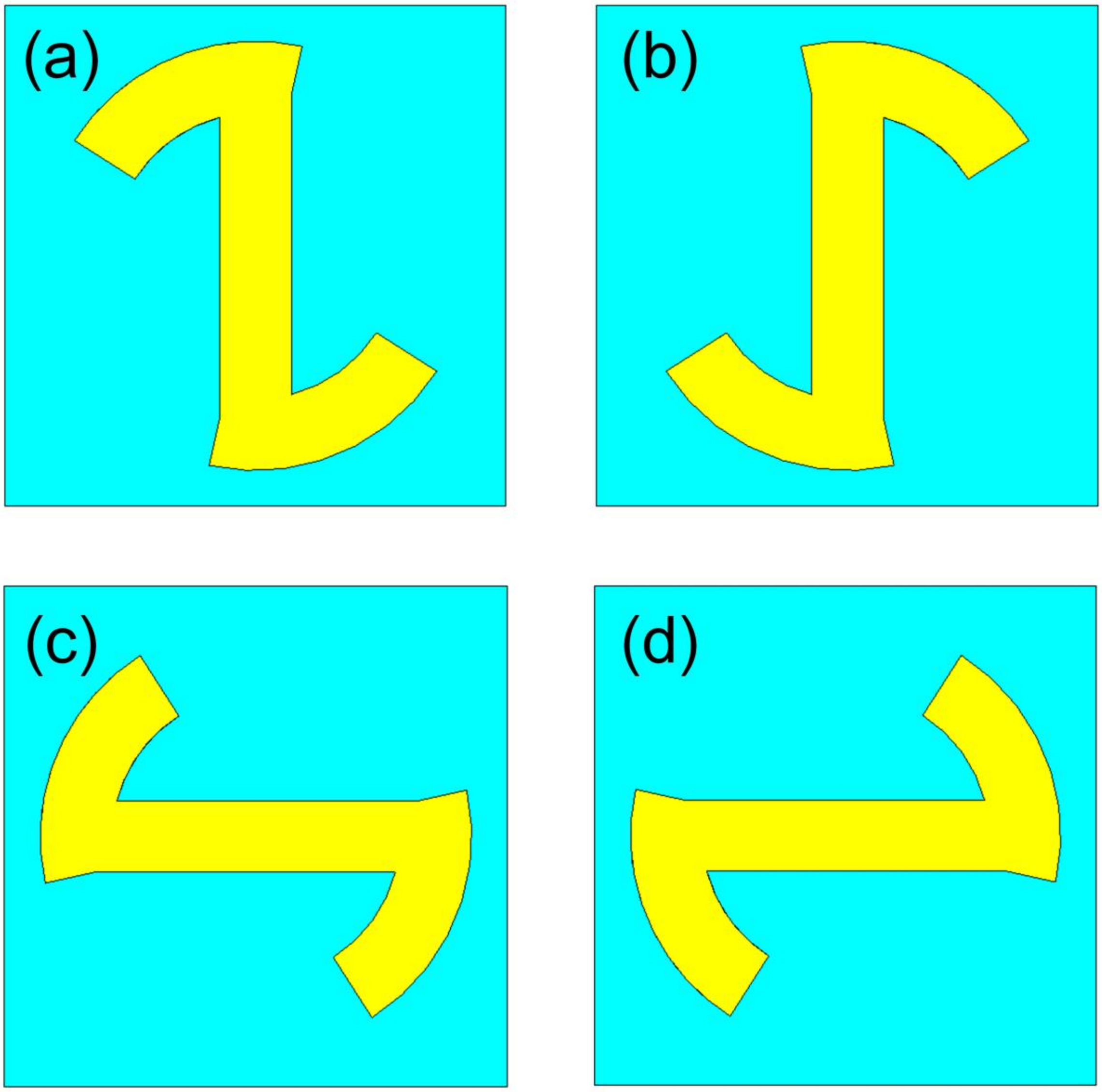

Figure S5. The geometric configuration of the meta-atoms, from which the non-separable coding states with one-to-one mapping correlation can be generated. (a) The structure is same with Sample # 2. (b) An axial symmetry transformation with the structure in (a). (c) Rotate the structure in (b) with τ=90°. (d) Rotate the structure in (a) with τ=90°.

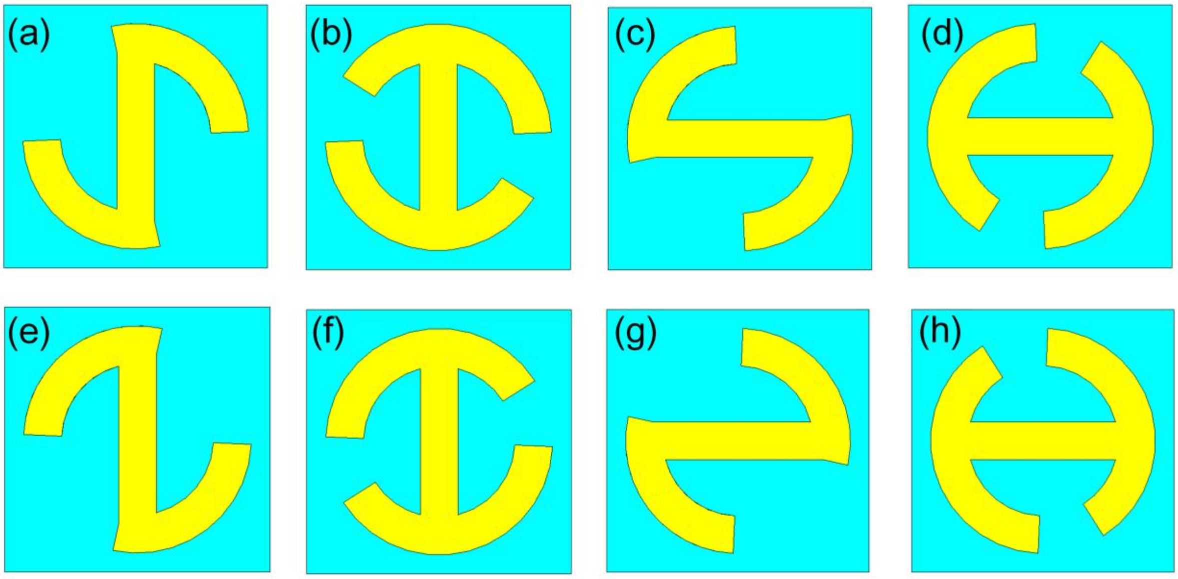

Figure S6. The two group of meta-atoms, which generate the non-separable coding states with one-to-multiple mapping correlation. The meta-atoms in (a-d) can generate 1-bit coding states on spin up, and 2-bit coding states on spin down. The meta-atoms in (e-f) can generate 2-bit coding states on spin up, and 1-bit coding states on spin down. (a, b) The spin-up trajectory remains at κ=175°, and spin-down path parameter evolves with γ=25° and γ=115°. (c, d) Rotate the structures in (a, b) with τ=90°. (e, f) The spin-down trajectory sets at γ=175°, while spin-up path parameter varies with κ=25° and κ=115°. (g, h) Rotate the structures in (e, f) with τ=90°. Obviouly, the structures in (a-d) are symmetric with that in (e-h).
